# Chicago Medical Society

**Published:** 1884-06

**Authors:** 


### CHICAGO MEDICAL SOCIETY.

The annual business meeting of this society was held on the evening of April 7, 1884, with the President, Dr. D. W. Graham, in the chair. Eighty-five members were present.

The annual reports of the Secretary, Treasurer, Committee on Library, and Auditing Committee, were read, received, accepted, and placed on file. Appended are the reports of each, in synopsis, after which election of officers for the ensuing year was held.

### SECRETARY'S ANNUAL REPORT FOR THE CURRENT YEAR ENDING APRIL 7, 1884.

*Mr. President, and Members of the Society:—*

While there may be nothing novel in my submitting to you a

*résumé* of that which goes to make up a report of the proceedings and business transacted in this society for the current year of 1883, it will, we trust, prove most gratifying and favorable to the members to be presented herewith a report which closes more than thirty years of our existence as an organization. During the year there has, probably, been a greater amount of more earnest work done than has ever before been performed by us. The duties of each one of you, it is presumed, have, to a certain degree and extent, been correspondingly increased. And I take great pleasure, therefore, this evening, in being able to make the very flattering statements about to follow, statements that in their simplicity may commend themselves to you; that the field gone over by us represents an advance that never before have we attained, so far as the knowledge of the present incumbent extends. This report will also contain a few figures for comparison regarding the business transacted in the society for the years 1882 and 1881, respectively.

During the year we have held 19 regular meetings and 1 special meeting.

Thirty-eight scientific essays and papers, written by 30 members, and a number of reports of cases, in writing, have been presented, with a larger number of pathological specimens than during any former year for a very long time, besides several nstruments of cure and mechanical appliances that have been exhibited.

Five hundred and four members and 134 visitors have attended 20 of the meetings, being in all 630 physicians and students, compared to 450 members and 120 visiting physicians, or 570 persons in attendance during 1882, and 304 members and 99 visitors, or 403 persons, in 1881.

The largest number in attendance at any of the meetings was about 100. The smallest number that attended any of the meetings is only 12. The average number of members in attendance at each meeting is $24\frac{14}{20}$ of guests $6\frac{14}{20}$; or a total of $31\frac{8}{20}$, compared to 30, the average number that attended the society the year previous, and 24, the average attendance during 1881.

Thirty-five new members have been received into active membership. (This number does not include the accessions, 8, this evening.) Compared to 34, the number admitted in 1882, and 18 elected to membership during 1882, or 87, the entire number received in the society during the three years of the present incumbent.

The membership list one year ago consisted of 210 members, and 181 constituted the number at the close of the year 1881. An increase of 35 during the year would make the total membership 245. Of this number, one has passed to the silent land, one resigned, and one left the city. This number of 3 deducted will leave a correct and accurate list of 242 members constituting this society. There are others who have removed from the city that wish to retain their membership, so that out of our total membership some 6 or 8 are non-resident members, of which a word upon this part of the report will be added later.

No less than 235 postal card announcements have been issued for each meeting, or a total number during the year of not less than 4,700. Regarding the time required to superscribe this number at the rate, computed approximately, of 40 postal cards an hour, it would take nearly 120 hours time to do this, which, at the rate of 10 hours a day, would be equivalent to 12 good days work for this single item alone to have been performed. Besides this, about 160–5 pages of records have been transcribed in this book, and a large number of communications answered. Your Secretary has also recently revised the membership list with the address of each member, and transferred the same, along with copying the Constitution and By-Laws, in the new book of records, which is the largest and finest we have ever had, and which I now show you.

The pages of records may, for sake of illustration, be compared to 86 and 61 pages, respectively, that were recorded during the two years previous. Perhaps no part of the records will find more ready appreciation than the faithful *verbatim* reports of the discussions. I say faithful, as implying as near as possible, that of a retentive memory, in addition to the comprehensive notes that were taken at the time of debate, and as discussion is clearly an important feature of our sessions, where they have appeared in print, an endeavor has been made to give to the profession everywhere most clearly the individuality of the members and the views of those who participated, without partiality being shown to anyone.

The list of papers includes a varied assortment of topics, the best that has ever been written by the members and presented here. In many of the papers there was so much value that they have been songht by other associations and re-read as well as being quoted, in every instance in abstract form or in their entirety, in several medical journals. And we trust the high standard in this respect will be maintained. The subjoined list of papers, including the author and date of reading, constitute this part of the year's summary.

(1) Prevention of Contagious Diseases and our Hospital Accommodations for Children. Dr. Frank E. Waxham, April 2, 1883.

(2) Treatment of Empyema. A paper prepared by Dr. E. F. Ingals, and read by Dr. W. L. Dorland, April 16, 1883.

(3) A Running Commentary on the Relation of Micro-Organisms to Disease, with an exhibition of 30 micro-photographs. Dr. W. T. Belfield, April 16, 1883.

(4) Exhibition of Dr. Wm. P. Verity's improved derrick and suspension apparatus for the application of plaster casts. Dr. Frank S. Johnson, May 7, 1883.

(5) Observations on the Published Proceedings of the Meeting of the Illinois State Board of Health, held at Chicago April 12–14, 1883. Dr. Ephraim Ingals, May 7, 1883.

(6) A Report on Recent Research in Operative Surgery. Dr. John E. Owens, May 21, 1883,

(7) "Stammering" is the subject of an amusing and instructive paper read by Dr. Alonzo Bryan, June 4, 1883.

(8) Exhibition of Bacteria under the Microscope, Removed from a Decayed Tooth. Dr. Robert Tilley, June 4, 1883.

(9) A paper on Obstetrics, including a report of a Trio of Triplets. Dr. Henry Ogden, June 18, 1883.

(10) How Doctors and Medical Societies should be Made More Efficient. Dr. Simon Strausser, July 2, 1883.

(11) Mechanical Equivalent to Animal Heat. Dr. H. D. Valin, July 16, 1883.

(12) Report of a Case of Bilateral and Forward Dislocation of the Fourth Cervical Vertebræ (wherein the patient recovered). Dr. W. L. Axford, July 16, 1883.

(13) A Written Report of a Case of Acute Hepatic Abscess, with Autopsy. Dr. C. T. Fenn, July 16, 1883.

(14) The special meeting, September 26, 1883, at which Sir Wm. MacCormaco addressed the society on several topics pertaining to the major operations in surgery, will again be alluded to in another part of this report.

(15) History of Insanity in Chicago, from an Analysis of 3,000 Cases. Dr. S. V. Clevenger, October 15, 1883.

(16) Report of Three Cases of Insanity from Quinine. Dr. J. G. Kiernan, October 15, 1883.

(17) The Effects of "Manganese" in Amenorrhœa and Menorrhagia. Dr. F. H. Martin, October 15, 1883.

(18) Is Extirpation of the Cancerous Uterus a Justifiable Operation? Dr. A. R. Jackson, November 5, 1883.

(19) Treatment of Trichiasis by Electrolysis. Dr. J. E. Colburn, November 5, 1883.

(20) The Immediate Operation for Laceration of the Perinæum. Dr. E. C. Dudley, November 19, 1883.

(21) Glycosuria. Dr. Oscar C. DeWolf, November 19, 1883.

(22) Treatment of Bright's Disease. Dr. Chas. W. Purdy, December 3, 1883.

(23) Treatment of Granulated Eyelids with Jequirity. Dr. F. C. Hotz, December 3, 1883.

(24) Report of an Obstinate Case of Sciatica, including the Details of Operation by Stretching the Sciatic Nerve, with Recovery. Dr. F. E. Wadhams, December 3, 1883.

(25) Alveolar Abscess Simulating Nasal Catarrh. Dr. John S. Marshall, December 17, 1883.

(26) Packing-House Wounds. Dr. Wm. L. Axford, December 17, 1883.

(27) Perverted Sexual Instinct. Dr. J. G. Kiernan, January 7, 1884.

(28) Potts' Disease, (The Mechanical Treatment of Dorsal Caries with Great Deformity, and Exhibition of Apparatus.) Dr. C. E. Webster, January 7, 1884.

(29) Gelseminum Sempervirens. Dr. Charles G. Davis, January 21, 1884.

(30) Report of a Case of Cystic Growth in the Posterior Nares, with Operation for Removal and Final Favorable Result, paper prepared by Dr. E. F. Ingals, read by Dr. Robert Tilley, January 21, 1884.

(31) Paretic Dementia in Females, with a report of a case. Dr. S. V. Clevenger, February 4, 1884.

(32) Treatment of Stricture of the Urethra. Dr. Henry J. Reynolds, February 4, 1884.

(33) Report of a Case of Ventral Hernia. Dr. Wm. L. Axford, February 18, 1884.

(34) Report of Three Cases of Arrested Fœtal Development., or Maternal Impressions. Dr. Luke A. Harcourt, February 18, 1884.

(35) Potts' Disease as Illustrating the Principles of Early Diagnosis. Dr. C. E. Webster, March 3, 1884.

(36) Report of a case of Aneurismal Tumor from the Arch of the Aorta (with specimen). Dr. O. J. Price, March 3, 1884.

(37) On the Ætiology of Typhoid Fever. Dr. J. F. Todd, March 17, 1884.

(38) Select Topics in the Surgery of the Nervous System, subdivided—I. Suture of Nerves. II. Elongation of Nerves. III. Exploration of the Brain by the Hollow Needle, paper prepared by Dr. Roswell Park, and read by Dr. L. H. Montgomery, March, 17, 1884.

A number of important committees have been appointed during the year, one of the most important of which is the Committee on Library. A review of their work has also been embodied in this report (for fear, perhaps, that they would not be present or prepared), but I notice its chairman present, and you will therefore hear from him in detail a more interesting report than this one. Twenty delegates from this society attended the annual meeting of the American Medical Association in Cleveland, last year, beside other permanent members of the Association from this society that attended, swelling the number to thirty-four. A slight digression for a moment, yet apropos to the subject, will be pardoned, when I allude to an extract from a letter recently received from the Permanent Secretary of the Association. He states: "The present membership is about 3,000, of which 1,100 attended the meeting one year ago. Your society did well," and this year if the twenty-five delegates to be elected to-night can attend the coming meeting of the Association to be held in Washington the first Tuesday in May next, assuming that the great distance will prevent more than thirty from attending altogether, who are members of this society, it certainly is to our credit, if the entire number in attendance at the Association meeting should consist of one-thirtieth of its members from Chicago. We hope also that all who can do so will attend the meeting of the State Society to be held in this city, beginning on the 3rd Tuesday of May next.

To recapitulate. One special meeting was held for the informal reception of a distinguished Fellow, Sir William MacCormac, with the number of regular meetings alluded to and regular order of scientific business. Well may we have reason to feel proud of the honor to be a member of the Chicago Medical Society, and our half dozen colleagues who are not resident members, some of whom are located in cities of other states, I believe desire to retain their membership here, if for no other reason, as two have informed me, than that of receiving the anticipated postal card announcement.

Regarding discipline and courtesy to each other, and to myself particularly, during the year just elapsed, I can bear testimony that a great deal of it has been extended, and it is to your interest as well as having the best interests of the society at heart, that this fraternal feeling for each other should long continue to exist, and of which the success this society has achieved is greatly due, and before closing I desire to add:

Be pleased to accept my thanks, especially for the many personal favors bestowed, and if mistakes have occurred, as they most surely will now and then, do not attribute it wholly to the officers, but extend your leniency to other causes and imperfections in proportion to the great pleasure the duties of the work have been performed by the Secretary; and for fear, possibly, of being misunderstood, I wish to state again, our success has been greatly aided by your united efforts, and with this I may say officially to you "vale!"

LISTON H. MONTGOMERY.

65 Randolph St., April 7, 1884.

---

The following is a synopsis of the voluminous report of the Committee on Library, which is also their first annual report, submitted by Dr. Edmund Andrews, Chairman:

| | |
|---|---|
| Money appropriated by the society | $500.00 |
| Money contributed by physicians, dentists, and druggists | 317.00 |
| Money pledged by four gentlemen to meet transportation of books from Europe and other expenses | 50.00 |
| 110 volumes of books, donated by various parties, estimated cash value | 155.00 |
| Total | $1,022.00 |

Of the cash above stated we have expended the following sums:

| | |
|---|---|
| Paid W. T. Keener, of which $500 was paid by the Treasurer of the society according to the appropriation | $528.15 |
| Paid for postage both ways, on circulars sent to 1,500 men | 63.00 |
| Paid for stationary and printing | 15.45 |
| Paid for express charges and sundries | 2.50 |
| | $609.10 |
| Which sum deducted from $817, the amount actually collected, as above stated, leaves cash on hand | $207.90 |

This latter sum, with the $50 pledged by four gentlemen, will more than cover the cost and transportation of books, which we have ordered from Europe.

Several well-known works are omitted, because new editions are about to appear, which will be best to purchase next year. By careful economy of the funds, we have been able to purchase the great French work, called the Dictionaire des Sciences Medicales; 125 "half volumes" are now out, and the rest will soon be issued. This magnificent work will give us the best thoughts of the French profession on every medical and surgical topic.

Then a list numbering 149 volumes, showing the titles of the works purchased and ordered, was read—also a list of eleven of the representative medical journals purchased.

Also a list of medical and surgical works donated by the following persons, valued at least to be worth $155.

Mrs. George C. Clark, 62 bound volumes.
W. T. Keener, 3 bound volumes.
Dr. R. B. Treat, 9 bound volumes.
Dr. R. Ludlam, 9 bound volumes.
Park, Davis & Co., 10 bound volumes.
Dr. A. B. Stockham, 3 bound volumes.
Dr. A. H. Foster, 3 bound volumes, worth at present, $30.
Dr. Justin Hayes, 1 bound volume.
Dr. H. P. Merriman, 6 bound volumes.
Anonymous Donor, 2 bound volumes.
Dr. E. Andrews, 3 bound volumes.

Summary of volumes:

| | |
|---|---|
| Bought of W. T. Keener | 160 vols. |
| Ordered from Europe, but not arrived | 74 vols. |
| Books donated | 110 vols. |
| Total | 344 vols. |

We find an increasing interest and enthusiasm on this subject on the part of the profession, and plans have been laid before us for raising very much larger sums annually for a period of years. The authorities of the Public Library coöperate with us more cordially, and are now diligently at work cataloguing and shelving the books preparatory for use. In accordance with the advice of this society, which we heartily endorse, the Librarian will keep them always ready for reading in the library and not loan them out. Very respectfully submitted,

E. ANDREWS, Chairman,
F. C. HOTZ,
OSCAR C. DEWOLF, Committee on Library.

Per L. H. MONTGOMERY, Secretary.

The following officers were elected for the ensuing year:

Dr. D. A. K. Steele, President.

Dr. Chas. W. Purdy, 1st Vice-President.

Dr. Curtis T. Fenn, 2d Vice-President.

Dr. Liston H. Montgomery, Secretary (re-elected.)

Dr. E. Fletcher Ingals, Treasurer, (re-elected.)

Dr. Wm. E. Quine, Dr. G. C. Paoli, Dr. D. R. Brower, Committee on Membership.

Dr. D. W. Graham was elected to the vacancy on the Committee on Library, caused by the expiration of Dr. Oscar C. DeWolf's time, as provided in the By-Laws.

Fifty dollars was voted unanimously to the Secretary, Dr. L. H. Montgomery, as a testimonial and souvenir for duties he had performed as an official during the year.

Fifty dollars was also voted unanimously to Messrs. John B. Drake & Co., proprietors of the Grand Pacific Hotel, for use of their parlors and other courtesies extended to the society.

Fifteen dollars was also voted unanimously to the Treasurer, Dr. E. F. Ingals, for losses he may have made good (as per discrepancies) in his annual report, as per Auditor's report.

Considerable miscellaneous business was rapidly dispatched, including a complimentary vote of thanks to the retiring President.

Dr. W. H. Curtis moved that this society do now adjourn, and the society at a late hour stood adjourned.

The following delegates from the Chicago Medical Society were elected at the annual meeting, held at the Pacific on the evening of April 7, 1884, to the American Medical Association, which will meet at Washington, May 6 next:

Dr. F. M. Wilder, Dr. A. Reeves Jackson, Dr. W. W. Allport, Dr. Eggleston Burrows, Dr. N. E. Rice, Dr. Frank Billings, Dr. J. G. Kiernan, Dr. E. F. Gaston, Dr. J. F. Todd, Dr. W. T. Montgomery, Dr. W. E. Clarke, Dr. C. S. DeVeny, Dr. Simon Strausser, Dr. C. J. Simons, Dr. A. H. Cooke, Dr. Philip Adolphus, Dr. J. E. Walton, Dr. T. W. Brophy, Dr. J. S. Marshall, Dr. H. J. Reynolds, Dr. L. H. Montgomery, Dr. J. H. Chew, Dr. E. F. Ingals, Dr. T. W. Miller, Dr. C. T. Parkes, Dr. E. J. Doering.

AUDITOR'S REPORT.

Dr. Curtis T. Fenn, Chairman.

1. Expenditures.—All expenditures were attested by proper vouchers, except $3 for clerical labor and $7.60 collectors' fees.

2. Receipts.—On comparing the cash list with the membership register and schedule of annual dues, it appeared that three ordinary members were on the one hand credited with dues which did not on the other hand enter into the list of cash received.

The omission was accidental, and partly due to disputes concerning the terms of the fiscal year. All questions of this kind have been, happily, removed.

3. Amount on hand at the beginning.—This part of the report it has been difficult to verify. The society has not required its reports to be audited, and for five years there has been no auditing committee until now.

That committee were, therefore, invited to take the books and to go through them searchingly and thoroughly. In glancing back, it was noticed that an entry in the cash account bearing date November 1, 1880, To balance, $2.65, re-enters in balancing August 4, 1881, causing an error, the correction of which will be $2.65 in favor of the Treasurer for the year ending April, 1882.

But there were also slight discrepancies in 1882 between the schedule of dues and the cash list, three persons having received credit and no corresponding entry having been made of the amount.

These three, although unable to show receipts, had refused to accept repeated duns, and the Treasurer on his part, believing the dues to be unpaid, had simply refused to charge the amount to himself. However, in view of the continued assertion of these members of their recollection of settlement, the Treasurer has made the books tally by taking the amount, as he believes, from his own pocket.

From 1882, the examination led to 1881, beyond which the committee did not find it convenient to go.

In 1881 sixteen persons are credited with dues whose names do not figure in the cash list. But it was in that year that Treasurer Davis died. The following note from the present Treasurer sufficiently explains itself.

" In looking over my ledger, I find that on the 9th of October, 1880, Dr. N. S. Davis handed to me all that was left at the time of the collections since the annual meeting, amounting to $17.40. I have no way of telling what the expenses of the society had been during those six months, neither are there any records to show how much the former Treasurer may have had on hand at the beginning of the fiscal year.

" Therefore I see no possible way of making the register before 1881 tally with the cash book, and as several of the dues for 1880 were paid in 1881, and there is no way of telling in most instances for what year any special payment should apply, I do not think it possible to make 1881 and the cash book tally. It will not be possible accurately to ascertain even about 1882."

From this may be seen what lay before the Auditor. One or two matters of simple criticism may be mentioned. The Treasurer's report of 1881 ends with $173.67 in the treasury. The report of 1883 begins with $156.32.

Again, the report of 1882 ends with $346.07, while the report of 1883 begins with $339.07.

These discrepancies in the reports themselves are explained by the fact that the present Treasurer was in the habit of including receipts and expenditures up to the close of the annual meeting in the year that had just preceded them.

There is one more item of some importance. Of thirty-three new members in 1883, there were fifteen at the end of the year who had not paid the initiation fee. These were all enrolled and the money in the treasury, therfore, there should have been $15 more (by Article V, Section 2 of the By-Laws) in the treasury.

Of seventeen new members in 1882, only twelve paid their initiation fee, making an obvious loss of $5 in the report of that year.

Returning to the matter in hand, this examination finds $6 to be added in 1883, and $6 in the previous year.

But $2.65 have to be subtracted in 1882. Moreover, in that year there was one payment of annual dues credited to "unknown," which must be subtracted, in all $4.65, leaving $1.35.

This and $6 makes $7.35 to be added in favor of the society. Instead, therefore, of $547.82 the true balance to 1884 becomes $550.06.

Respectfully submitted,

CURTIS T. FENN, Chairman.<br>

LISTON H. MONTGOMERY, Secretary. } Auditing Com.

This report was, upon motion, duly seconded, received, accepted, and placed on file.

